# Can an Amine Be a Weaker and a Stronger Base at the Same Time? Curious Cases of Chameleonic Ionization

**DOI:** 10.1021/acsphyschemau.3c00029

**Published:** 2023-08-31

**Authors:** Robert Fraczkiewicz

**Affiliations:** ‡ Simulations Plus, Inc. 42505 10th Street West, Lancaster, California 93534, United States

**Keywords:** Ionization constants, protonation, multiprotic, prediction, macrostate, microstate, fraction ionized

## Abstract

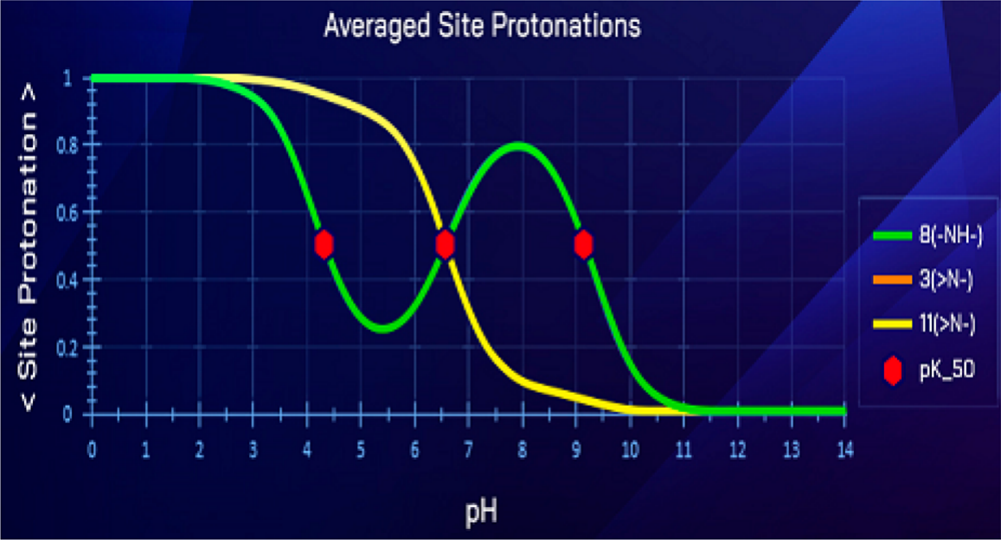

We discovered an anomalous basic dissociation in certain multiprotic compounds. An amine group placed in the middle of a given compound is predicted to behave unusually—at certain pH ranges, its averaged degree of protonation actually *increases* with pH (!) resulting from interactions with other ionizable groups. This chameleonic behavior results in two p*K*_50_ values: one corresponding to a weaker base and the other to a stronger base for the same group.

Predictive models of protic ionization constants that accurately take a complete thermodynamics of ionization into account, such as our S+p*K*_a_,^1^ must necessarily predict all microequilibria. Experimental determination of ionization microconstants is possible, but it requires sophisticated techniques, it is limited to simple compounds, and it is not routinely done.^2^ It is unfortunate since newly developed concepts of averaged single-proton acidity (ASPA), averaged site protonation (ASP), and single-proton midpoint (p*K*_50_)^3^ allow a chemist to gain valuable insights into complex ionization phenomena of multiprotic compounds and quantify the precise role of ionizable functional groups. In particular, for a given ionizable group, *G*, the averaged site protonation, is defined as the following pH-dependent ratio:
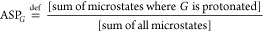
The pH at which the ASP profile crosses 50% is recognized as an intrinsic “group p*K*_a_” and is named the single-proton midpoint (p*K*_50_). For “clean” ionization macrostates that are strongly dominated by a single microstate (i.e., by a state that defines ionization of a particular functional group), the group’s p*K*_50_ is close to the corresponding macroscopic (i.e., apparent) p*K*_a_ measured in standard titration experiments. Otherwise, the p*K*_a_ results from ionization of many groups (i.e., multiple microstates), and such a simple correspondence does not exist. All computational details of the predictive modeling methodology and ASP/p*K*_50_ calculations have been published in open access articles.^1,3^

Soon after we had implemented^4^ the aforementioned concepts, we discovered rare cases of unusual ionization. Normally, the ASP profile of a multiprotic compound follows the expected titration-like behavior: All site protonation curves decrease with the pH (Figure 1). This is expected since with dropping concentration of hydronium ions in water the protonation of ionizable sites, regardless of type, should strictly decrease, as well.

**Figure 1:**
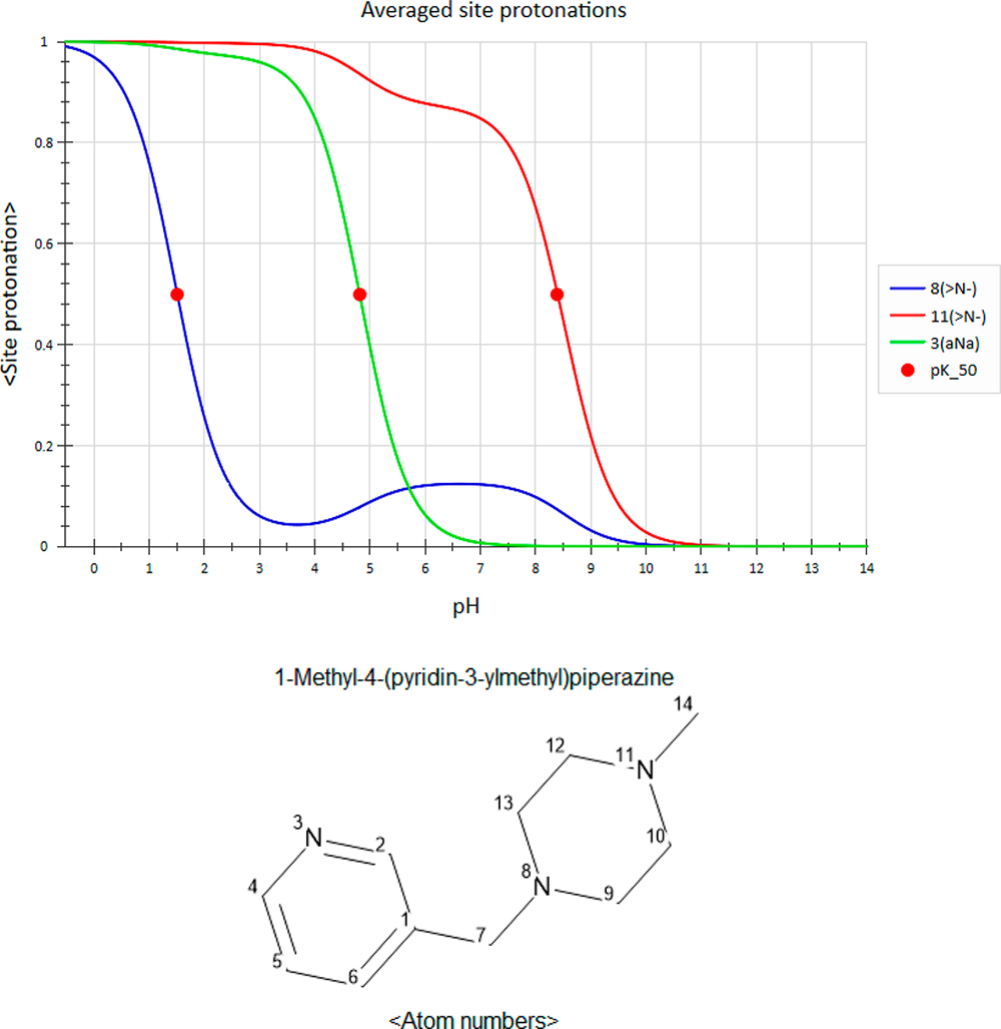
Predicted^4^ ASP profiles and atom numbering of 1-methyl-4-(pyridin-3-ylmethyl)piperazine. The p*K*_50_ values are 1.51 (atom 8), 4.81 (atom 3), and 8.39 (atom 11).

To our surprise, in rare cases, the averaged site protonation of certain amines were predicted^4^ to actually *increase* with pH! At first, we saw this anomalous behavior in molecules with at least three amine groups, such as indinavir (an HIV protease inhibitor,^5^Figure 2). In a stunning turn of events, the population of the drug’s middle amine (atom 8, blue curve) *gains* protons from pH = 3 up to pH = 5.5! Closer examination of the drug’s ionization microstates points to a plausible physical mechanism. The indinavir eight microstates are shown in Figure 3. It is easier to understand protonation patterns while going from high to low pH by, e.g., imagining a titration of the compound with a strong mineral acid. In such a titration, the middle amine is protonated first. Indeed, the corresponding microstate dominates the HM^+^ macrostate at 79.2%. For comparison, the peripheral amines (atoms 3 and 11) contribute only 5.1% and 15.7%, respectively. Hence, at sufficiently high pH, the middle amine is the strongest base. However, with decreasing pH the emerging HM^+2^ macrostate is dominated (84.7%) by a microstate where the two protons actually reside on the peripheral amines. Why? One might think that it is a significantly different drop in microconstant values on going from +1 to +2 macrostate: While the microconstants of the middle amine drop by 2–3 log units, depending on the final microstate, both peripheral microconstants drop by only ∼0.5 log units (Figure 3). It is clear that the proximity and interactions of middle and peripheral amines drop the basicity of the former more substantially than when only peripheral amines are protonated. However, as Figure S 1 in the Supporting Information shows, the microconstant drops for 1-methyl-4-(pyridin-3-ylmethyl)piperazine are similar in analogous transition: The middle amine microconstants drop by 2–4 log units and peripheral microconstants change by ∼0.5 log units. And yet the ASP plot for this compound does behave the way indinavir ASP does (although there is a small bump in atom 8’s curve between pH 4 and 9; Figure 1).

**Figure 2:**
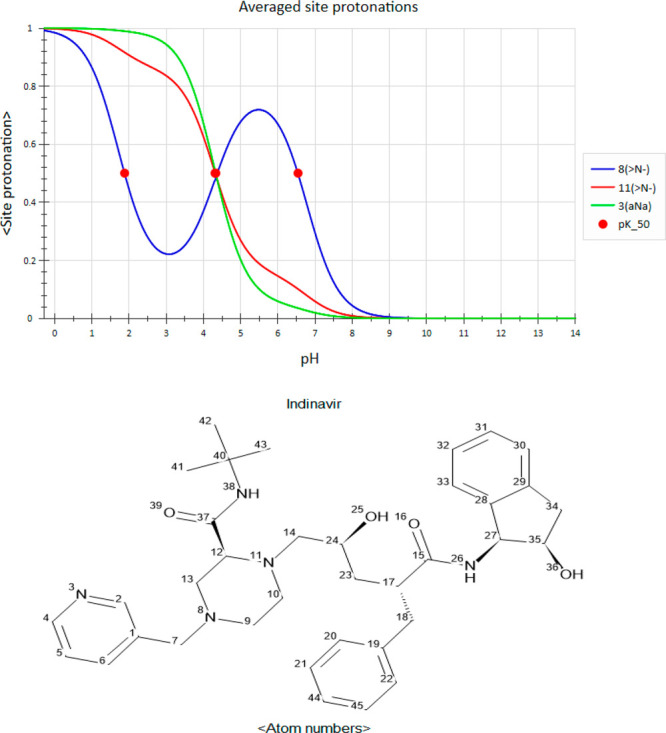
Predicted^4^ ASP profiles and atom numbering of indinavir (an HIV protease inhibitor^5^). The p*K*_50_ values are 1.88 and 6.54 (atom 8), 4.33 (atom 3), and 4.32 (atom 11).

**Figure 3:**
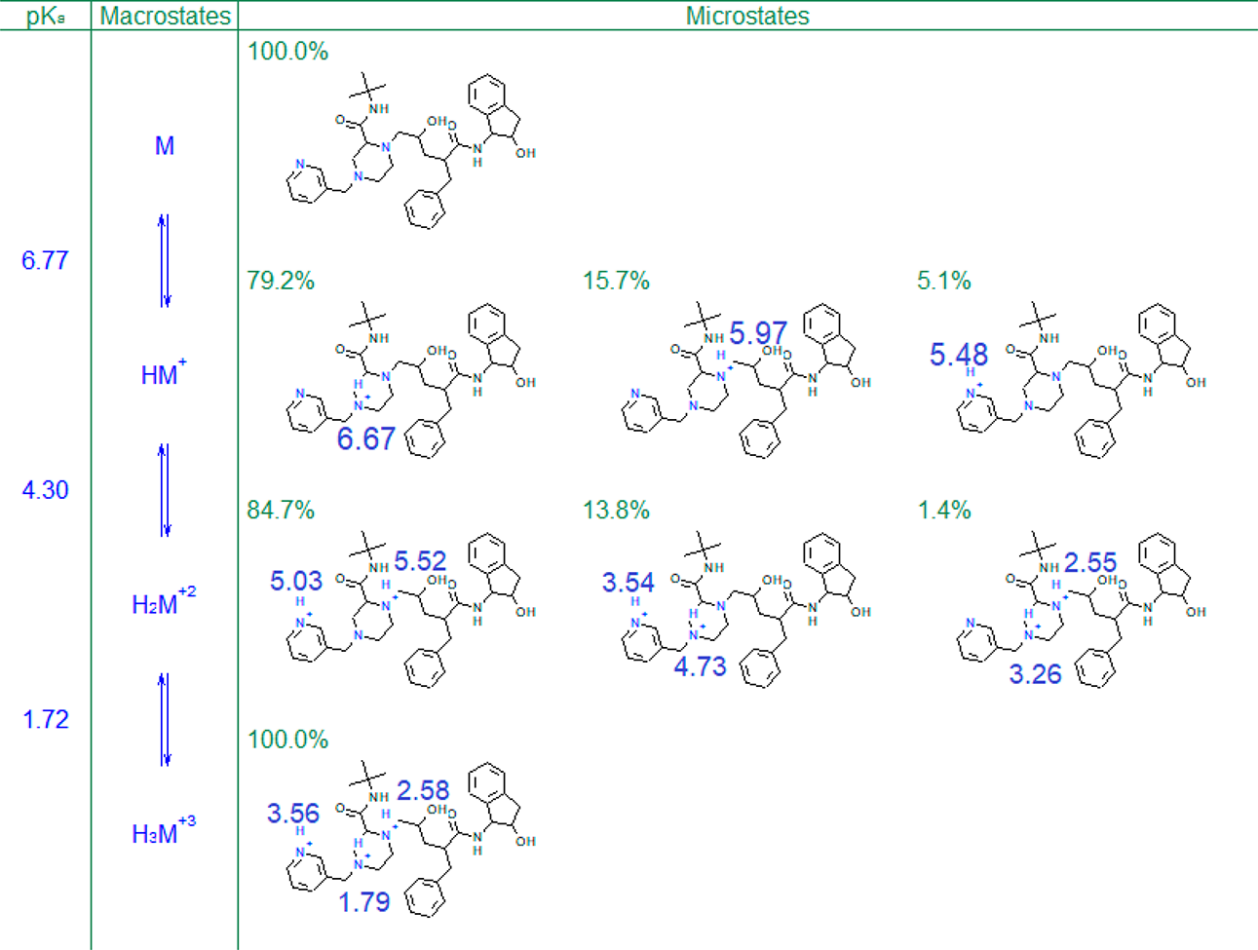
Predicted^4^ ionization microstates and microconstants of indinavir.

The question remains: Why are the two ASP profiles so remarkably different? The answer lies in the relative basicities of atom 11 in both compounds: It stands out as the strongest base in 1-methyl-4-(pyridin-3-ylmethyl)piperazine (it dominates the HM^+^ macrostate). In contrast, the basicities of all three amines in indinavir are similar, ensuring stronger interactions between them. This point is best illustrated by pASPA profiles that plot each group’s averaged acidity (i.e., conjugate basicity) as a function of pH^3^ (Figures S 2 and S 3). The red curve in both plots represents the basicity of atom 11. While it runs above pASPA profiles for atoms 3 and 8 in 1-methyl-4-(pyridin-3-ylmethyl)piperazine, it is enveloped by curve 8 in indinavir. It seems the nearby N-t-butyl amide substituent is responsible for significant lowering of atom 11’s basicity as illustrated by the ASP profile for a suitable molecular fragment of indinavir (Figure S 4). Indeed, the p*K*_50_ of fragment’s atom 11 (4.95) is only about half of its counterpart in 1-methyl-4-(pyridin-3-ylmethyl)piperazine (8.39, Figure 1). The likely mechanism is the inductive effect of the proximal amide.

It is thus the indinavir middle amine deprotonation–protonation–deprotonation–protonation sequence in the M–HM^+^–HM^+2^–HM^+3^ macrostates, respectively, caused by strong interaction between individual groups that is responsible for its up-and-down ASP profile. Both peripheral amines, in contrast, follow the “traditional” deprotonation–protonation pattern and their ASP profiles are titration-like. Looking at this compound as a whole, there seems to be nothing anomalous: its global Bjerrum plot is monotonically decreasing (Figure S 5).

In ref (3), we introduced the concept of p*K*_50_, the midpoint of an ASP profile, as an indicator of functional group’s true basicity in a multiprotic compound. The ASP curve of the middle amine of indinavir crosses the 50% protonation threshold at three points with pH values of 1.88, 4.37, and 6.54 (Figure 2). However, only two of these, 1.88 and 6.54, are on the downward slope of the profile and as such correspond to protic dissociation fulfilling the spirit of the p*K*_50_ definition. The middle amine is therefore both the weakest (1.88) and the strongest (6.54) basic group in this compound—a truly chameleonic behavior! The remaining midpoint of this group, 4.37, is on the upward slope of its ASP curve and thus reflects protic *association* of this group; we do not count it as a p*K*_50_. Interestingly, this almost coincides with the p*K*_50_ values of both peripheral amines.

In the case of multiple p*K*_50_ values, which one should be used in research? It depends on the application. If our example compound is studied at human blood pH of 7.4, then 6.54 should be used since it is the first microstate of the HM^+^ macrostate that dominates at this pH. Conversely, 1.88 becomes important in, e.g., human stomach pH of 1.2 (where HM^+3^ dominates).

In conclusion, it is a subtle interplay of sufficiently close local basicities that triggers the chameleonic ionization in some compounds. We have extracted a number of interesting examples from the World Drug Index (see the Supporting Information). It would be truly interesting to verify the predicted chameleonic ionization experimentally through well-designed ^15^N NMR titrations. Unlike monotonic profiles of ^15^N chemical shifts in function of pH observed for aminoglycosides,^6,7^ for example, such titrations performed for chameleonic drugs should be nonmonotonic as illustrated in our examples. We invite researchers with access to an NMR instrument to do so, particularly to study medically important drugs!

## Supporting Information Available

Predicted Bjerrum plot, predicted ASP and pASPA profiles, and atom numbering of additional example compounds (PDF)

## Notes

ADMET Predictor is available for evaluation and academic research purposes free of charge.
