# Supplementary material for: Can an Amine Be a Weaker and a Stronger Base at the Same Time? Curious Cases of Chameleonic Ionization
Source: ACS Phys Chem Au. 2023 Aug 31;3(6):512–4. doi: 10.1021/acsphyschemau.3c00029 (PMC10683473; doi:10.1021/acsphyschemau.3c00029)
Supplement: Supplementary file 1 — pg3c00029_si_001.pdf [file pg3c00029_si_001.pdf]

Supplementary Information

Can an Amine Be a Weaker and a Stronger Base at the Same Time? Curious Cases of Chameleonic Ionization.

Robert Fraczkiewicz

Simulations Plus, Inc. 42505 10th Street West, Lancaster, CA 93534, USA.

E-mail: robert.fraczkiewicz@simulations-plus.com

Figure S 1. Predicted[1] ionization microstates and microconstants of 1-methyl-4-(pyridin-3-ylmethyl)piperazine.

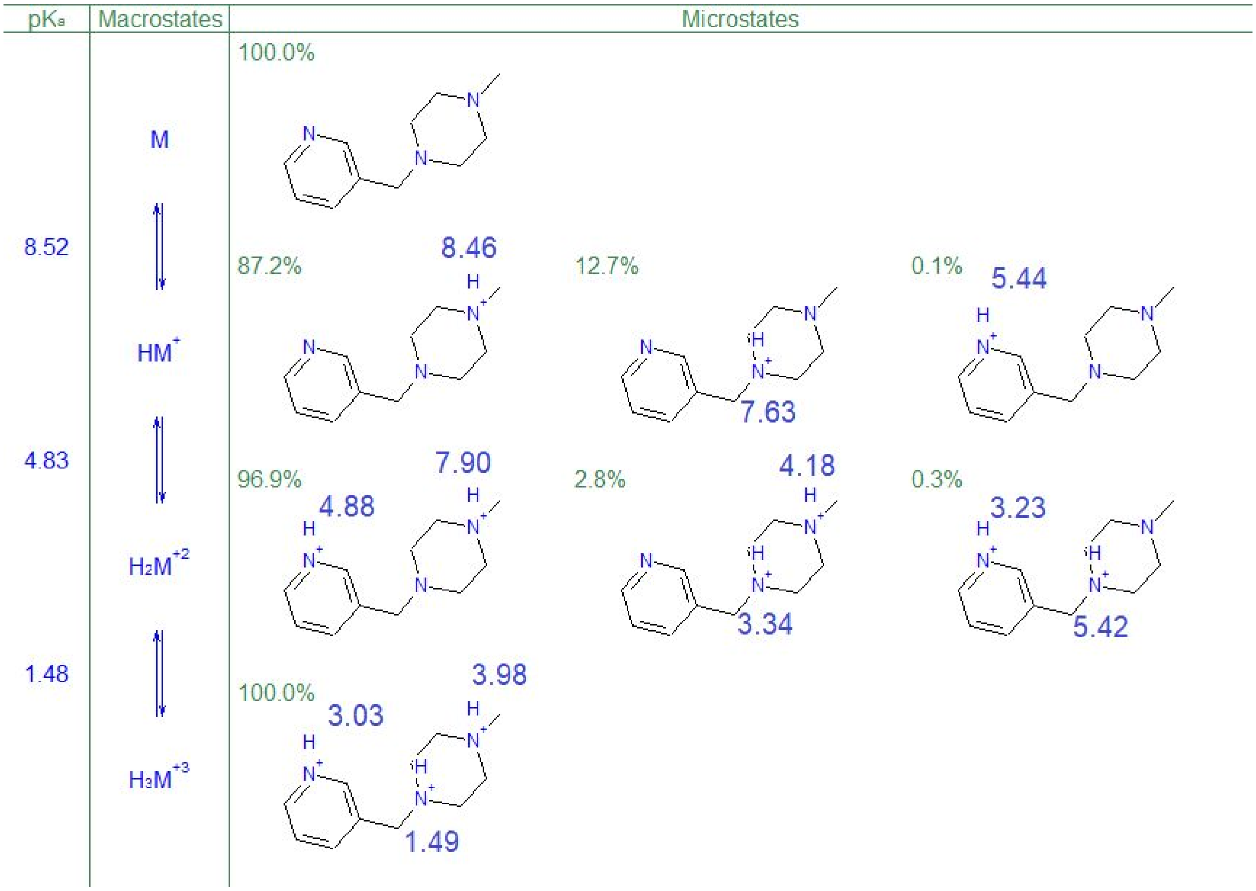

Figure S 2. Predicted[1] pASPA profiles and atom numbering of indinavir.

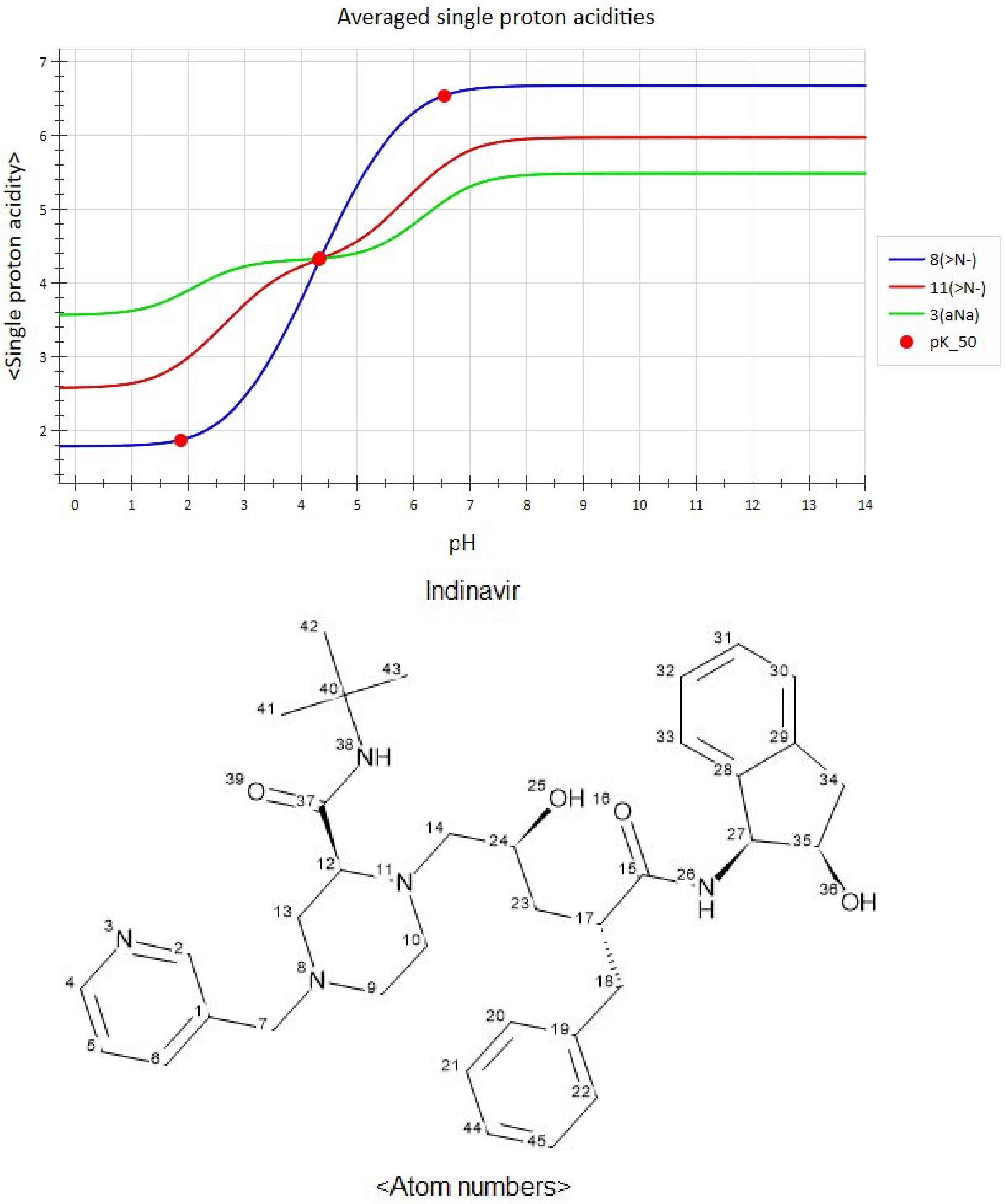

Figure S 3. Predicted[1] pASPA profiles and atom numbering of 1-methyl-4-(pyridin-3-ylmethyl)piperazine.

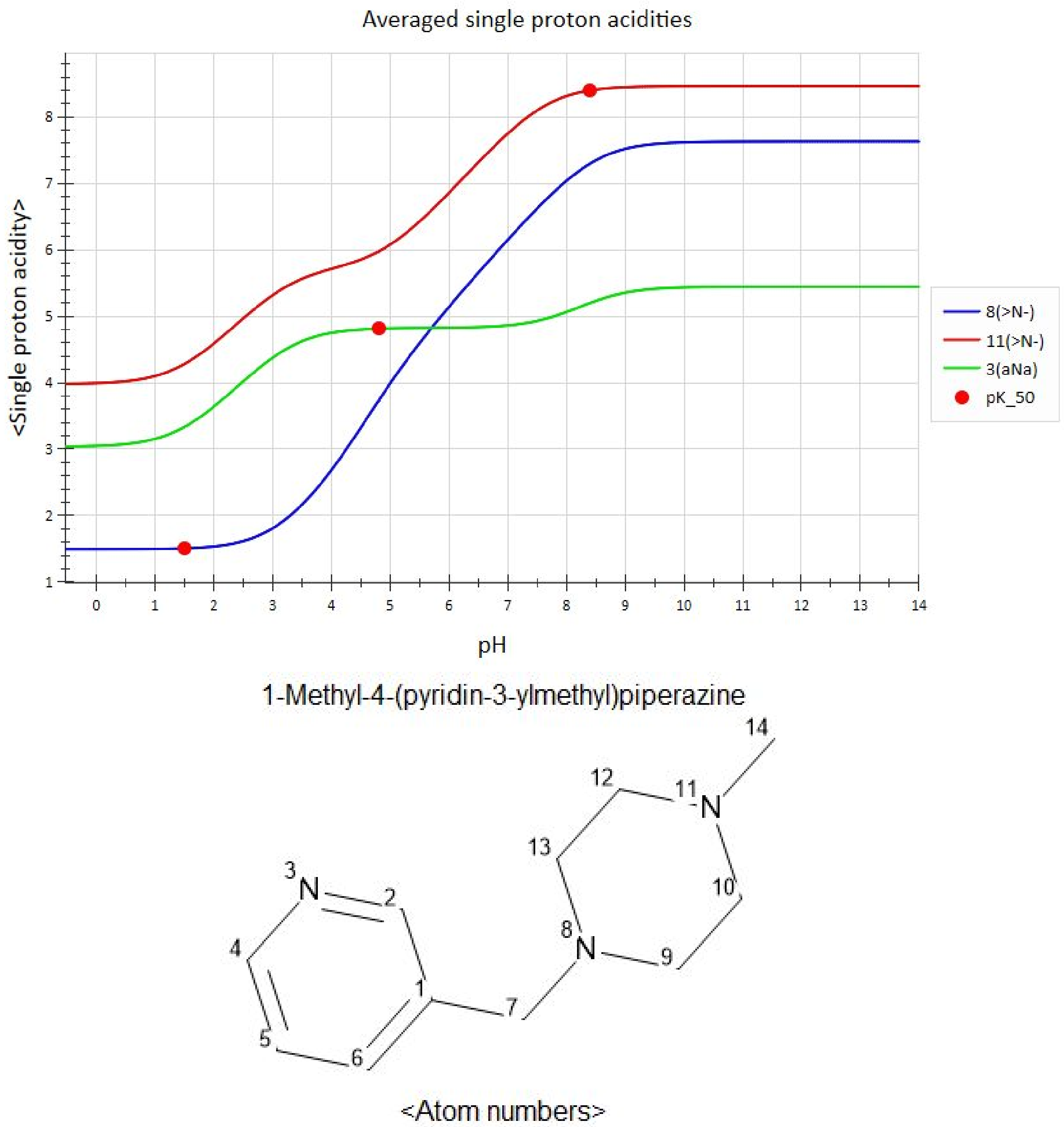

Figure S 4. Predicted[1] ASP profiles and atom numbering of an indinavir fragment described in text. The pK_{50} values are: 1.42 and 6.62 (atom 8), 4.52 (atom 3), 4.95 (atom 11).

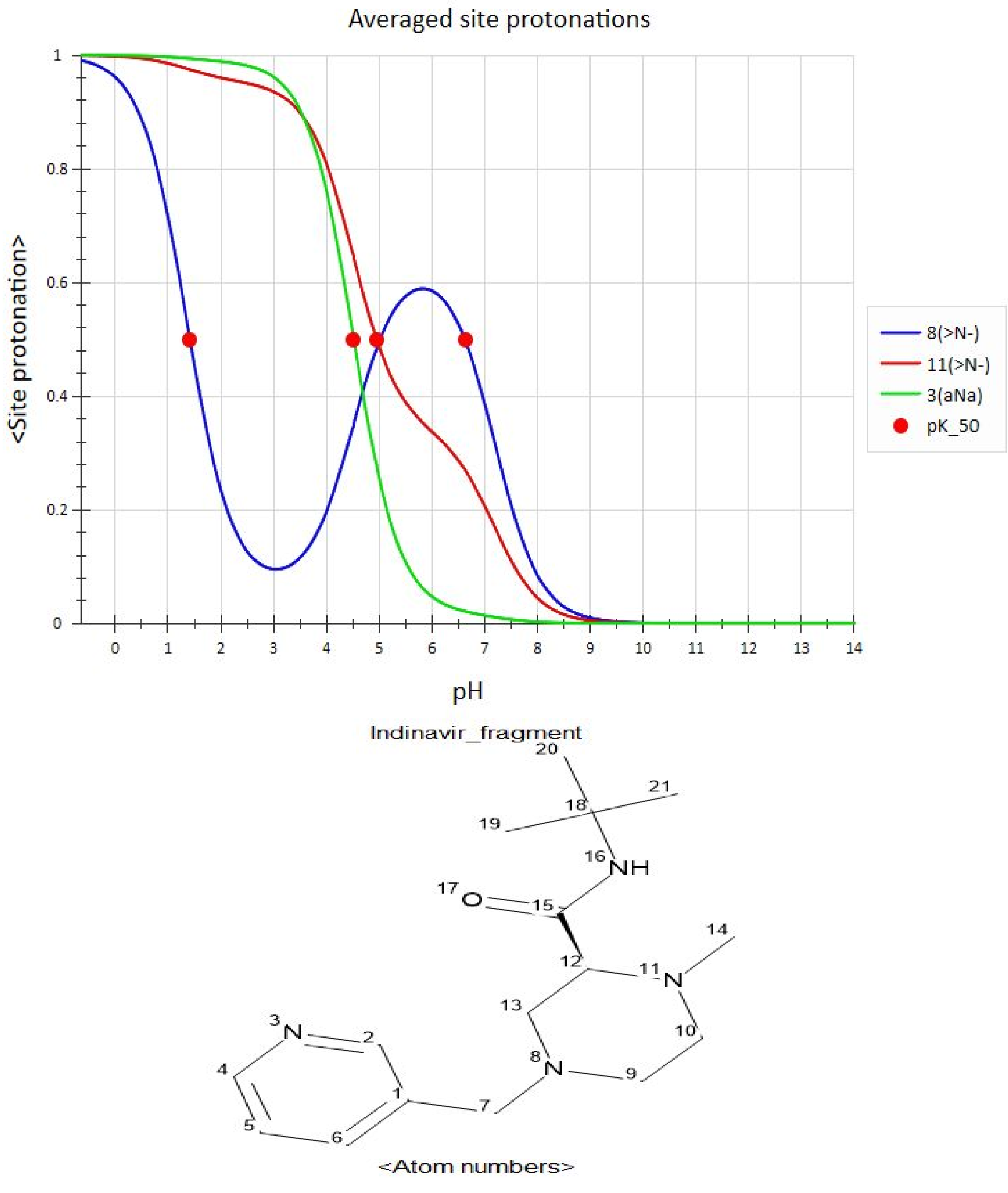

Figure S 5. Predicted[1] Bjerrum plot (global average number of protons as a function of pH) of indinavir.

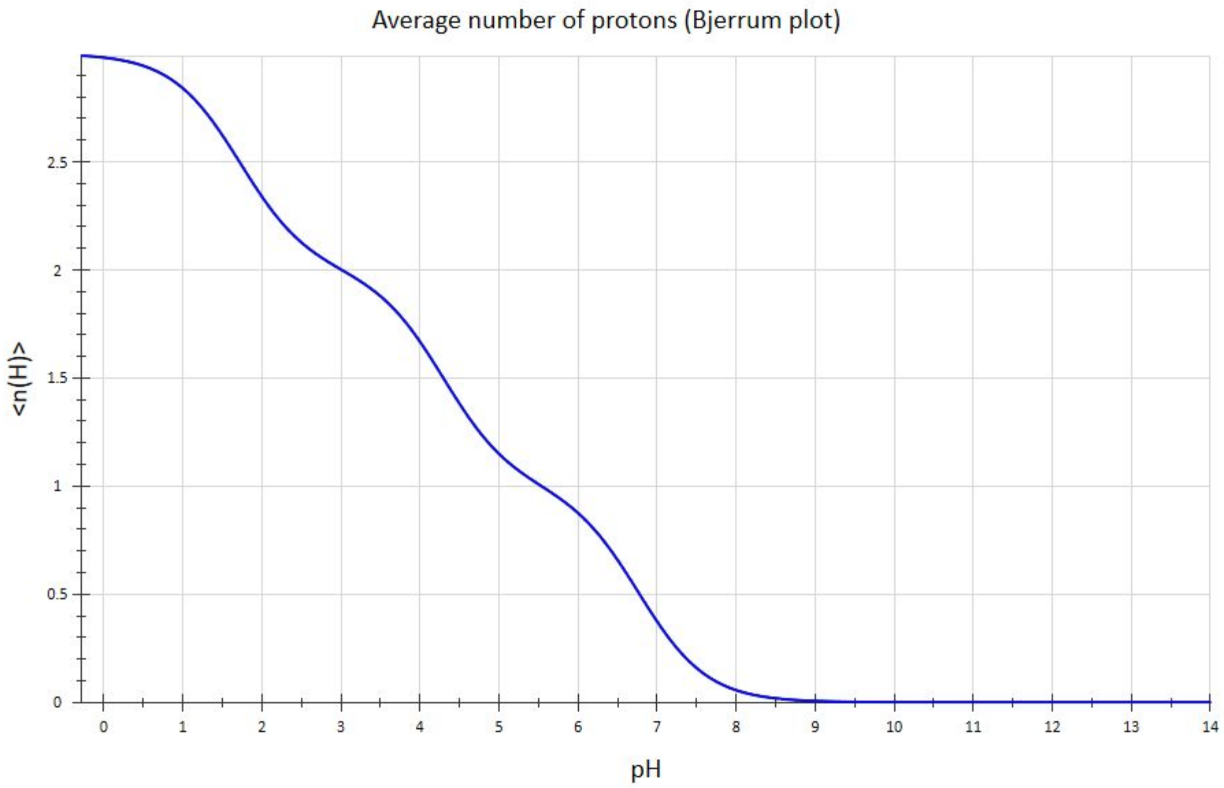

Average number of protons (Bjerrum plot)
<n(H)>
2.5
2
1.5
1
0.5
0
0
1
2
3
4
5
6
7
8
9
10
11
12
13
14
pH

Figure S 6. Predicted[1] ASP profiles and atom numbering of caldiamide (a gadodiamide[2] complexation ligand present in Omniscan formulation).

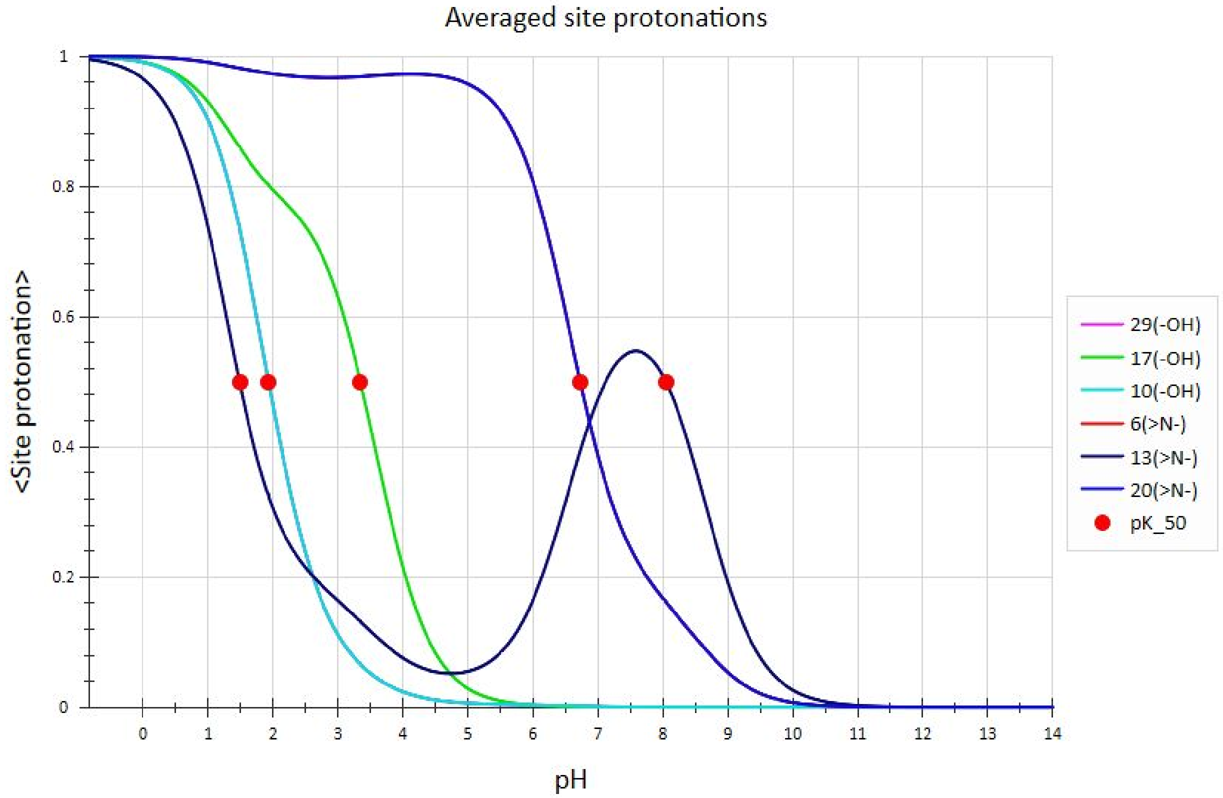

CALDIAMIDE

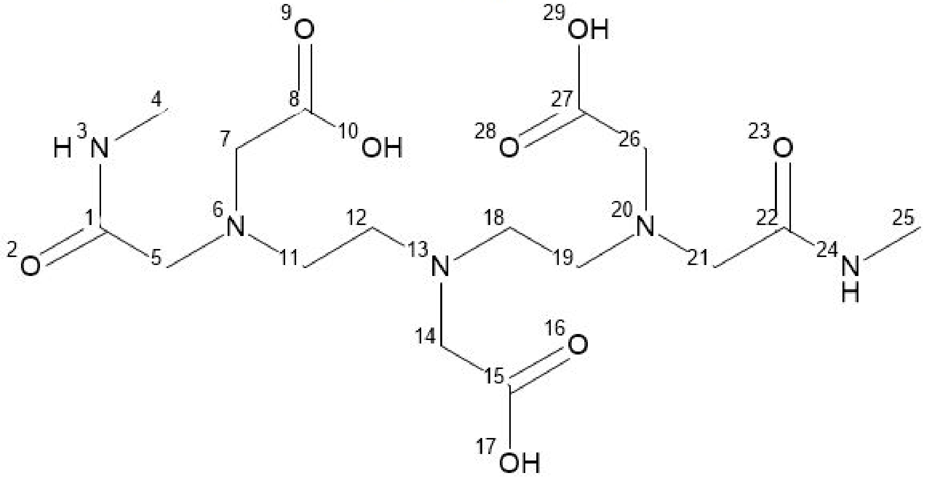

Figure S 7. Predicted[1] ASP profiles and atom numbering of dipicolinic acid (a.k.a. 2,6-pyridinedicarboxylic acid[3]). Curves for atoms 8 and 12 overlap perfectly.

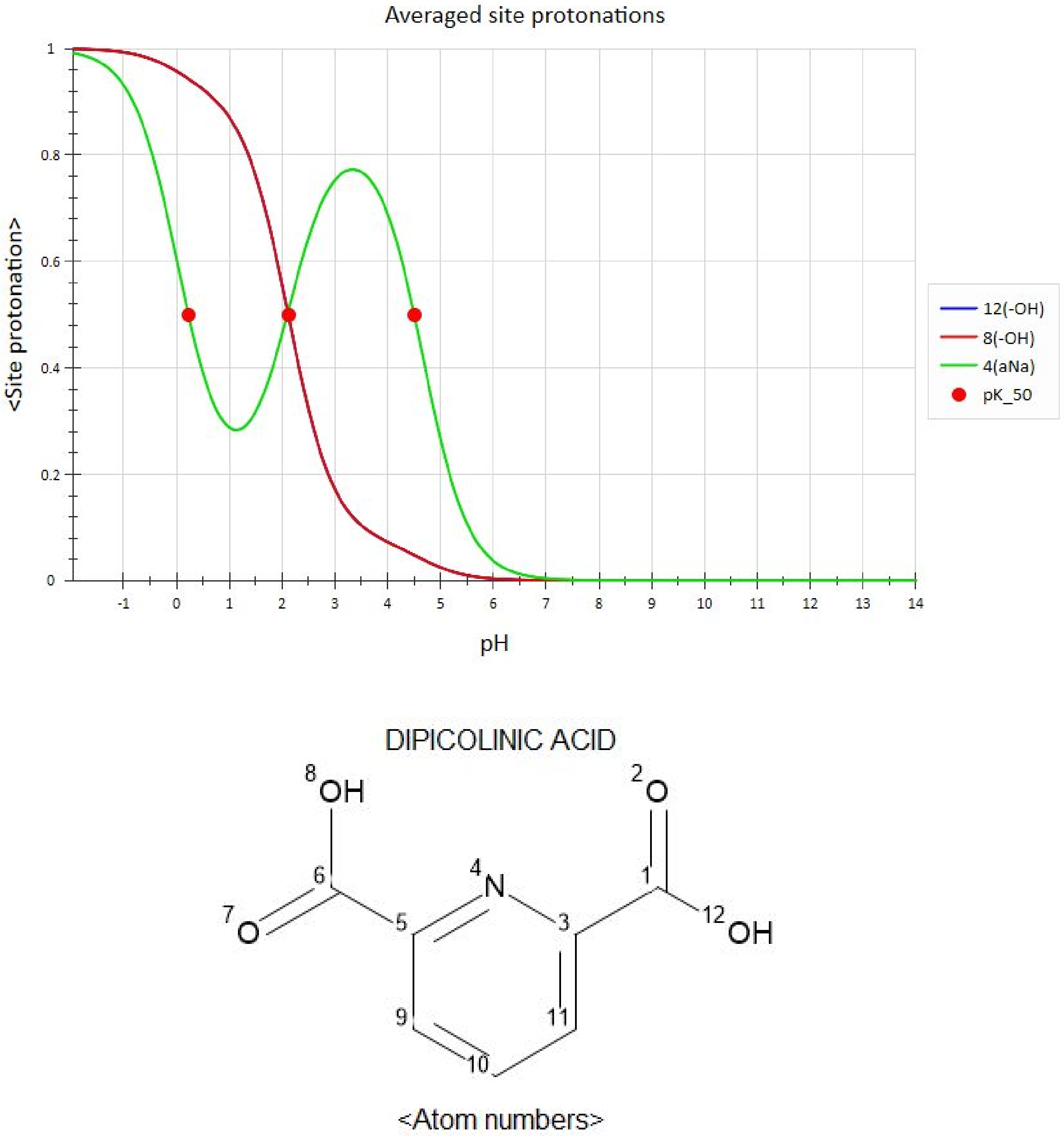

Figure S 8. Predicted[1] ASP profiles and atom numbering of FK-401 (an inhibitor of chymotrypsin[4]).

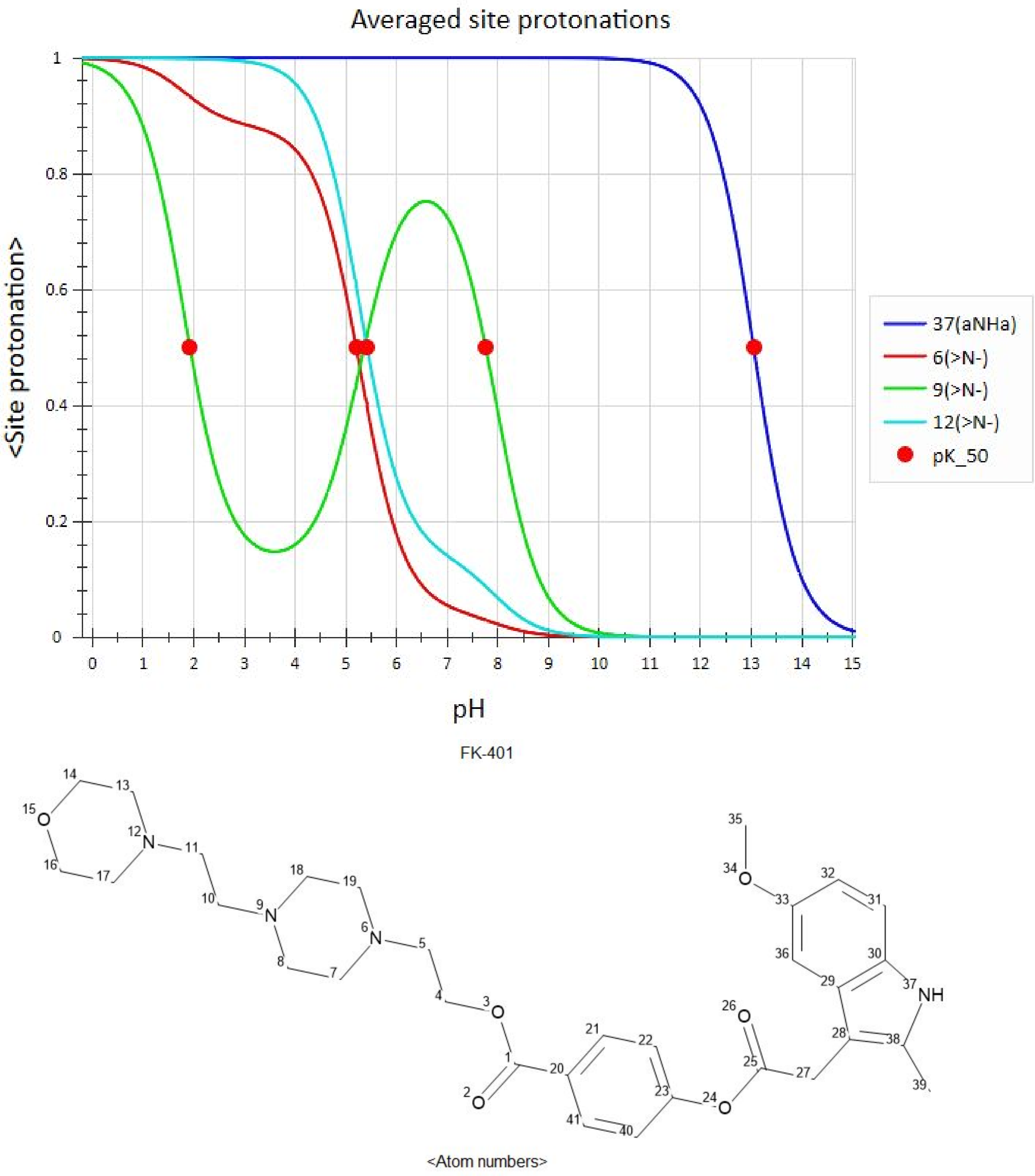

Figure S 9. Predicted[1] ASP profiles and atom numbering of jineol (a natural product investigated for potential medicinal applications[5,6]).

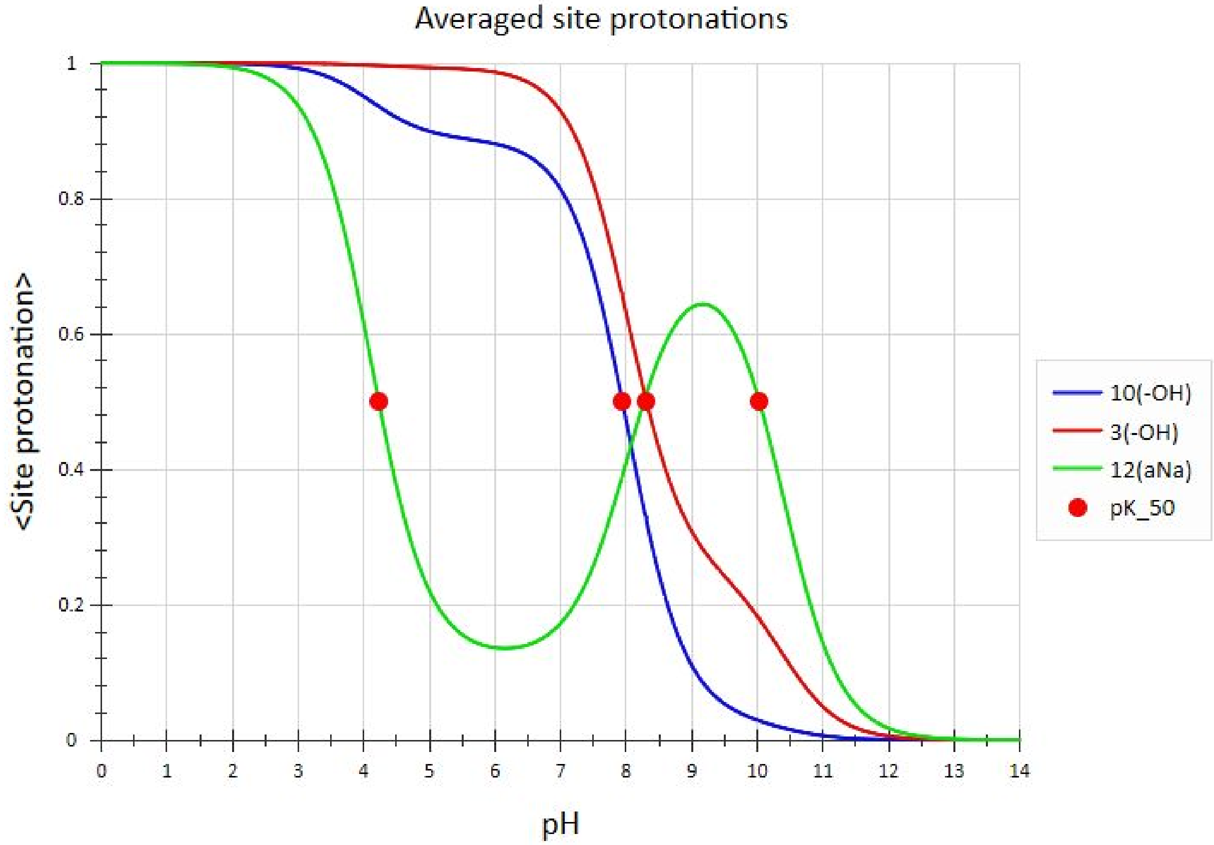

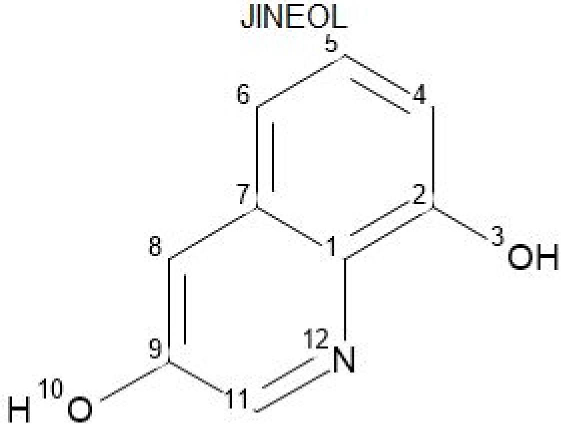

<Atom numbers>

References

(1) ADMET Predictor(R) *v 11.0*; Simulations Plus, Inc.: Lancaster, CA, USA, **2023**

(2) Gadodiamide. [Online Early Access]. Published Online: 2023. https://en.wikipedia.org/wiki/Gadodiamide (accessed 05/11/2023).

(3) Dipicolinic acid. [Online Early Access]. Published Online: 2023. https://en.wikipedia.org/wiki/Dipicolinic_acid (accessed 05/23/2023).

(4) Fujii, S.; Yokoyama, T.; Ikegaya, K.; Yokoo, N. New Synthetic Inhibitors of Chymotrypsin. *The Journal of Biochemistry* **1984**, *95*, 319.

(5) Bajpai, V. K.; Shukla, S.; Paek, W. K.; Lim, J.; Kumar, P.; Na, M. Antibacterial Action of Jineol Isolated from Scolopendra subspinipes mutilans against Selected Foodborne Pathogens. *Front Microbiol* [Online Early Access]. DOI: 10.3389/fmicb.2017.00552. Published Online: 2017. https://doi.org/10.3389/fmicb.2017.00552 (accessed 2017).

(6) Alam, M. B.; Bajpai, V. K.; Lee, J.; Zhao, P.; Byeon, J.-H.; Ra, J.-S.; Majumder, R.; Lee, J. S.; Yoon, J.-I.; Rather, I. A.; Park, Y.-H.; Kim, K.; Na, M.; Lee, S.-H. Inhibition of

melanogenesis by jineol from Scolopendra subspinipes mutilans via MAP-Kinase mediated MITF downregulation and the proteasomal degradation of tyrosinase. *Scientific Reports* **2017**, *7*, 45858.
